# Patient privacy and autonomy: a comparative analysis of cases of ethical dilemmas in China and the United States

**DOI:** 10.1186/s12910-021-00579-6

**Published:** 2021-02-02

**Authors:** Hui Zhang, Hongmei Zhang, Zhenxiang Zhang, Yuming Wang

**Affiliations:** 1 School of Nursing and Health, Zhengzhou University, No. 101, Science Avenue, Zhengzhou, 450001 Henan China; 2 Department of Scientific Research and Discipline Construction, Henan Provincial People’s Hospital and the People’s Hospital of Zhengzhou University, Zhengzhou, 450003 Henan China; 3 Nursing Department, Henan Provincial People’s Hospital and the People’s Hospital of Zhengzhou University, Zhengzhou, 450003 Henan China; 4 Clinical Research Centre, Henan Provincial People’s Hospital and the People’s Hospital of Zhengzhou University, No. 7, Weiwu Road, Zhengzhou, 450003 Henan China

**Keywords:** Patient privacy, Patient autonomy, Ethical dilemma case, China, The United States, Law, Culture, Bioethics

## Abstract

**Background:**

Respect for patients’ autonomy is usually considered to be an important ethical principle in Western countries; privacy is one of the implications of such respect. Healthcare professionals frequently encounter ethical dilemmas during their practice. The past few decades have seen an increased use of courts to resolve intractable ethical dilemmas across both the developed and the developing world. However, Chinese and American bioethics differ largely due to the influence of Chinese Confucianism and Western religions, respectively, and there is a dearth of comparative studies that explore cases of ethical dilemmas between China and the United States.

**Methods:**

This paper discusses four typical cases with significant social impact. First, it compares two cases concerning patient privacy: the “Shihezi University Hospital Case”, in which a patient was used as a clinical teaching object without her permission, and the “New York-Presbyterian Hospital Case”, in which the hospital allowed the filming of a patient’s treatment without his consent. Second, it compares two cases regarding patient autonomy and potentially life-saving medical procedures: the “Case of Ms. L”, concerning a cohabitant’s refusal to sign a consent form for a pregnant woman’s caesarean, and the “Case of Mrs. V”, concerning a hospital’s insistence upon a blood transfusion for a dissenting patient. This paper introduces the supporting and opposing views for each case and discusses their social impact. It then compares and analyses the differences between China and the United States from cultural and legislative perspectives.

**Conclusions:**

Ethical dilemmas have often occurred in China due to the late development of bioethics. However, the presence of bioethics earlier in the US than in China has not spared the US of ethical dilemmas. This paper highlights lessons and inspiration from the cases for healthcare professionals and introduces readers to the role and weight of privacy and autonomy in China and in the US from the perspectives of different cultures, religions and laws.

## Background

By the 1970s, with the joint promotion of technology and humanity, bioethics became a global phenomenon. It emerged in the United States [1], across Europe [2], and in China due to the “reform and opening-up” policy [3]. Bioethics is an emerging discipline that uses a variety of ethical approaches to study the philosophical, social, and legal issues arising in medicine and the life sciences in an interdisciplinary and cross-cultural context. It is intended to provide clinicians with a comprehensive framework for understanding the moral aspects of medicine [4].

Despite there has been controversies and challenges in relation to the four principles of bioethics—respect for autonomy, non-maleficence, beneficence, and justice—respect for the patient’s autonomy is usually considered to be an important principle in Western countries [5, 6]. To respect the autonomy of agents represents an acknowledgement of their right to hold views, make choices, and take actions based on their values and beliefs [7]. Invasion or infringement of privacy actively undermines individuals’ sense of self; therefore, privacy and confidentiality are important aspects of respecting people’s autonomy [8, 9]. Privacy contributes to the preservation of a sense of reverence and dignity; hence, it is vital for establishing and maintaining a respectful and effective clinical relationship [10, 11].

In Western society, moral precepts have their origins in both religion and philosophy [12]. The predominant religion of America—Christianity—is based on the idea that God (or some deity) reveals insights about life and its true meaning, prescribing clear and unambiguous rules that must be followed. Law shares four elements with religion: ritual, tradition, authority and universality. These four elements connect the legal order to beliefs in an ultimate, transcendent reality. Law provides religion with its social dimension while religion gives law its spirit and direction, as well as the sanctity it needs to command respect [13].

Conversely, traditional Chinese ethics focus on the responsibility of a person to work for the good of others [14]. The interdependence of family and community define the greater morals and social meanings in society, which overrides the concept of autonomy [15]. According to Confucianism, ethics is based on humanism; therefore, it does not include peremptory standards, codes, or norms that restrain people’s conduct. It encourages people to judge “right and wrong” based on their nous. Thus, unlike Western societies, it is difficult to find statutory moral codes in China.

Healthcare professionals frequently encounter situations that involve medical ethical principles leading to ethical dilemmas [16, 17]. In the past few decades, the use of courts, to resolve intractable ethical dilemmas, has increased across both developed and developing countries [18–21]. Despite the growing volume of literature exploring patient privacy and autonomy, there is a dearth of comparative studies that explore cases of ethical dilemmas between China and the United States. Consequently, the aim of this study is to bridge this gap and to consider the role and weight of patient privacy and autonomy from an intercultural and legislative perspective between the two countries.

## Methods

As mentioned above, this study aimed to conduct a comparative analysis of cases involving ethical dilemmas—from China and the United States—to understand the importance of patient privacy and autonomy. Four representative empirical cases (two from China and two from the US) are presented, with an analysis of the supporting and opposing views for each case. All names used in the cases were replaced with pseudonyms or initials, and the following sub-sections include illustrations of the cases.

### Privacy

#### Case 1: Shihezi University Hospital case

On September 15, 2000, A Jing, a 22-year-old unmarried patient, went to the First Affiliated Hospital of the Medical College of Shihezi University in Xinjiang for an abortion. When she laid on the bed undressed waiting for examination, the physician, Dr. A, abruptly summoned more than 20 people wearing white coats into the room. A Jing felt so embarrassed and ashamed that she asked the physician to make them leave, but the physician responded that it did not matter and that they were all interns. Then, Dr. A proceeded to explain the names of private body parts, early pregnancy symptoms, and examination procedures to the interns, a process which took about 5 min. A Jing was indignant and decided to protect her rights and interests through the legal system. On October 8, A Jing filed a lawsuit against the hospital in the People’s Court of Shihezi City, asking for an apology and compensation of RMB 10,000 (around $1200) for the mental distress that she had to endure. The court held a private hearing of the case on October 27 [22], and decided in favour of the plaintiff.

Arguments presented in favour of the hospital were as follows: first, bedside clinical teaching is an essential component of medical education [23]; training medical students is both a responsibility and a legal obligation of teaching hospitals. The majority of hospitals in China believed that patients automatically consented to serving as bedside clinical “teaching material” for trainees by seeking care at teaching hospitals. This notion led to the belief that informed consent was unnecessary. Second, medical students are not unnecessary personnel, but are both interns and future physicians; therefore, clinical teaching aims to educate the next generation of healthcare professionals rather than intentionally inflict harm upon the subject. Third, bedside teaching is required because it improves medical students’ skills, which benefits future patients. In Chinese culture, public interest outweighs personal interest, thus, patient altruism supersedes individual privacy, which is why it may be overridden in favour of bedside teaching.

Conversely, compelling arguments were also presented against the hospital. First, the obligation to teach legally binds the health administration offices, hospital, and medical students; however, this does not include patients. Hospitals and patients have equal legal status, sharing a fiduciary relationship that is contractual in nature. Therefore, patients are not obliged to cooperate with or participate in clinical teaching. Second, the involvement of students during an intimate examination of patients requires the patients’ explicit permission [24]. Conducting teaching activities after the patient has expressed their discontent and failing to obtain their informed consent for the same is a direct violation of the patient’s autonomy and privacy. Third, only physicians are professionally expected to keep the information that patients provide them or that they obtain during their professional interaction with patients confidential. Medical students do not have the professional qualifications to do the same; therefore, blurring the distinction between medical students and physicians is incorrect.

This was the first lawsuit in China in which a hospital was sued for causing mental anguish to a patient because her permission was not obtained during clinical teaching. This sparked a heated societal discussion and raised patients’ consciousness regarding safeguarding their rights. Since then, there have been additional cases of patients defending their privacy across China. At that time, there was no concept of privacy rights or penalty clauses in China’s law. The right of privacy was protected by the right of reputation indirectly; the court considered acting on the violation of privacy only if the plaintiff’s reputation was also violated or affected. The main reason that many patients’ privacy rights have been arbitrarily violated is due to inadequate legislation. Case 1 (in 2000) and a series of subsequent cases led to legislative reform. In 2002, China started to review the draft Tort Liability Law, which was finally enforced on July 1, 2010. In China’s legislative history, the concept of “privacy right” was established for the first time in the Tort Liability Law, and came to be seen as an independent right of personality. Disclosing other people’s privacy, even if it does not infringe upon their reputation, constitutes infringement. Consequently, health management departments have accelerated their procedures on the protection of patient privacy. Medical institutions have been working toward making suitable adjustments to modify their infrastructures and regulations as well as to provide humanistic medical training to staff and interns.

#### Case 2: NewYork-Presbyterian case

In August of 2012, Mrs. C, a 75-year-old woman, saw a medical documentary filmed by The American Broadcasting Company (ABC) called “NY Med”. She recognized Dr. B, a surgeon who had tried to save her husband’s life 16 months prior. In the documentary, the surgeon was treating a man injured in a car accident. Although the image was blurred, the man could be heard in the episode. Mrs. C recognized that the man was her husband, Mr. C. In 2013, Mr. C’s family sued ABC, New York-Presbyterian (NYP) and Dr. B for violation of patient privacy. After 3 years of litigation, on April 21, 2016, the Department of Health and Human Services’ Office for Civil Rights (OCR) announced a $2.2 million settlement agreement with NYP [25].

The arguments that were presented in favour of the hospital follow. First, NYP decided to take part in the reality series to help educate the public about the challenges faced by medical professionals and the complexities of medical care. Second, ABC claimed that the medical documentary inspired people to go to medical school, to seek treatment they otherwise would not have known about, or to become organ donors, and because “NY Med” was produced by its news division, it was protected by the First Amendment. Third, ABC blurred the victim’s face and used voice alteration software to obscure any potential identifying information; therefore, the hospital’s duty to avoid disclosing personal information was not breached [26, 27].

The cogent countervailing arguments included the following. First, all of the intimate details of a person’s health are supposed to be shared only with the patient and whoever else they designate, however, the crew was allowed to film Mr. C’s treatment at the hospital and broadcast the moments leading up to his death, without the patient’s or his family’s permission. Second, patients and their families are vulnerable, and the TV show caused Mr. C’s family emotional distress and psychological harm. Medical ethicists and groups such as the American Medical Association worry that such shows exploit the suffering of patients for public consumption. Third, although the hospitals and doctors did not receive money in return for allowing the filming of their procedures, but they did benefit in the form of free publicity [27, 28].

Consequently, litigation has pushed for stricter regulations. It was decided that it is not sufficient to film patients and then obtain consent to broadcast the material, nor is it permissible to use blurring, pixilation, or voice alteration to mask the identities of patients whose consent has not been obtained. In addition, NYP initiated a corrective action plan to update policies and procedures and to develop workforce training. It also had to be monitored by OCR for two years to ensure compliance with the Health Insurance Portability and Accountability Act (HIPAA) obligations while it continued to provide care for patients [25].

#### China versus the United States from a cultural perspective

As a common phenomenon, privacy exists in various cultures. In ancient times, people covered their bodies with leaves and hides, suggesting that the earliest understanding of privacy related to the private parts of the body. In Case 1, the patient sued the hospital because of an invasion related to her private body parts. It indicated that consciousness of patient privacy was slow to evolve and that the concept of patient privacy was relatively weak in China. The term “privacy” was translated from English and introduced to mainland China after the 1980s. The translation of “privacy” in Chinese is *yinsi*—the word *yin* means “hide” and the word *si* means “private” or “privacy” and has long been used as an antonym to *gong* (public) and *guan* (official). Historically speaking, China has always treated individuality with suspicion and has advocated for collectivism and cohesion for centuries. Common people regard mutual care and assistance as a symbol of unity and harmony, and believe that privacy is primarily associated with internal secrets or scandals related to personal reputation. Most people think that if their private matters becomes public, their reputation will be tarnished. Thus, the term “privacy” has a negative connotation. It represents a scenario in which the focus on individual ownership of private information is bleak and shielding one’s reputation in the community is more prominent [29].

On the contrary, American culture takes greater pride in individual accomplishments and enjoys more personal freedoms and privacy than collectivist cultures. Merriam-Webster defines privacy as “the state or condition of being free from being observed or disturbed by other people; the state of being free from public attention”. This definition is broader than the concept of privacy in Chinese culture. The right to privacy is considered a fundamental right in a free society; it is a notion that runs deep in American culture, and is to be both respected and defended.

#### China versus the United States from a legislative perspective

The famous article published in the Harvard Law Review in 1890 by Warren and Brandeis on the “Right to be let alone” marked the birth of privacy protection in the US [30]. In 1965, the Supreme Court case Griswold v. Connecticut established the right to privacy as a constitutional doctrine [31]. A variety of laws have worked in tandem to allow Americans to stand up for their privacy rights, such as the Family Educational Rights and Privacy Act of 1974, the Americans with Disabilities Act of 1990, and the Health Insurance Portability and Accountability Act (HIPAA) of 1996. Since its enactment (a period of over two decades), HIPAA has been extremely successful in accomplishing its primary objective: making patients feel safe giving their physicians and other treating clinicians sensitive information while permitting reasonable information flows for treatment, operations, research, and public health purposes [32].

In China, the legal protection of privacy rights started late; from the 1980s to 2010, privacy protection was included in the right of reputation protection in China. On July 1, 2010, China’s Tort Liability Law came into effect, which for the first time explicitly stipulated that there is a “right of privacy” in China. The formal recognition and protection of privacy rights in the Tort Liability Law has brought about great changes in legal practice; privacy right has been shifted into an independent reason of tort. Nevertheless, the definition of the right of privacy was not clearly defined, and proper operation of the law requires further elaboration. Meanwhile, as society develops and technology advances, people’s privacy content also expands. On May 28, 2020, the third session of the 13th National People’s Congress passed the Civil Code. Privacy is here defined for the first time, and it is defined as a natural person’s sphere of life protected from interference, where a person should be left in peace, as well as the sphere of information and actions that individuals do not want to be of public domain and hence not shared with other people. This was the first law that was defined as a “code” that enriches the connotation of privacy and the scope of applicable protection [33].

### Autonomy

#### Case 3: the case of Ms. L

On November 21, 2007, Ms. L, a 22-year-old woman who was in her ninth month of pregnancy, was sent to Beijing Chaoyang Hospital by her cohabiter, Mr. X. The physicians suggested an immediate caesarean operation because the patient had developed severe pneumonia. However, Mr. X insisted for a normal delivery stating that Ms. L just had a cold. The hospital learned that the pair was suffering economic hardships; consequently, they offered to perform the operation free of charge. Ms. L went into a coma because her situation had worsened, thus, her partner Mr. X was asked to sign the surgery consent form. Doctors had spent three hours explaining the situation and persuading Mr. X, but he ultimately wrote “refuse the caesarean section, and bear the consequences” on the form. Ms. L and the unborn child both died despite continuous efforts to save them. On January 24, 2008, Ms. L’s parents filed a civil lawsuit against the hospital. In December 2009, the Court of First Instance ruled that Chaoyang Hospital had not caused any infringement, and the hospital decided to give the plaintiff 100,000 yuan (around $14,500) on humanitarian grounds. However, the plaintiff refused to accept this judgment and lodged a second appeal. On April 28, 2010, the Court of Second Instance dismissed the appeal and upheld the original judgment [34].

Arguments presented against the hospital are as follows. First, respecting the sanctity of life should be paramount and the physicians’ actions violated the right to life. They did not follow Article 24 of the Law of Medical Practitioners, which states that emergency measures should be taken for diagnosis and treatment of patients in critical condition. Second, Article 33 of the Administrative Regulations on Medical Institutions stipulates that in cases of special circumstance, the physician shall propose a medical plan and implement it after obtaining the approval of the leader or authorized personnel of the medical institution. Third, Article 33 of the Regulations on the Handling of Medical Accidents clearly stipulates that emergency measures undertaken to save the life of critically ill patients, but that cause adverse consequences, shall not be regarded as medical malpractice, thus, physicians should have performed the surgery without obtaining informed consent from the patient or her cohabitant.

Conversely, the hospital presented the following arguments. First, the physicians had taken emergency measures apart from surgery; they had urgently asked the superior department for instructions to operate without the family’s signature. In addition, because it was difficult for ordinary people to understand Mr. X’s behaviour, the hospital had invited the director of the neurology department to assess his mental state. The police were also contacted to search for Ms. L’s family members with whom she had been disconnected for years. Second, Article 33 of the Administrative Regulations on Medical Institutions clearly stipulates that the hospital must obtain the consent of the patient and the signature of their family members or legal acquaintance before an operation. The regulation of “special circumstances” in Article 33 is vague; some scholars believe that it refers primarily to patients whose identity is not known, or who have no family members or source of income. Third, the physician–patient relationship is a civil legal relationship. According to Article 4 of the General Principles of Civil Law, civil activities should comply with the principle of voluntariness; therefore, because Mr. X’s signature expressing his explicitly disagreement with the operation, if doctors forced to the caesarean, the hospital will bear legal liability in case of an accident during the surgery.

This case caused a furore amongst the media and the public. While most netizens believed that Mr. X should be held responsible for the tragedy, nearly all official media insisted that the hospital should have treated the patient without consent. Additionally, many scholars proposed to amend the relevant laws and regulations. At the end of 2007, the emergency disposal rights of medical institutions were written in the draft of the Tort Liability Law. Article 56 of the Tort Liability Law (2010) officially stated, “if the opinions of patients or their close relatives cannot be obtained due to emergency situations such as rescuing patients in critical condition, corresponding medical measures can be adopted immediately with the approval of the leader or authorized person of the medical institution”. Some legal professionals termed it the “Mr. X clause”.

#### Case 4: the case of Mrs. V

On August 27, 1994, a pregnant woman, Mrs. V, gave birth to a healthy baby at the Stamford Hospital in Connecticut, US, but she endured heavy bleeding due to placenta residue. A blood transfusion was proposed to save her life; however, Mrs. V and her husband refused the procedure because it was against their religious beliefs. Before undergoing any procedure, she executed a release requesting that no blood or its derivatives be administered to her during her hospitalization, Mrs. V’s husband also signed the release. Despite the fact that Mrs. V would die without blood transfusions, she and her husband maintained their decision. Her obstetrician, Dr. C, tried dilation, curettage, and other alternatives that did not require blood transfusion, but her condition continued to deteriorate. On August 28 at 2 a.m., Dr. C submitted a request to the court regarding Mrs. V’s case, which resulted in the trial court eventually permitting the hospital to administer blood transfusions. Mrs. V was then given blood transfusions and recovered from the complication, but she appealed to the Appellate Court contesting the trial court’s judgment. On April 9, 1996, the Supreme Court of Connecticut ruled that Stamford Hospital violated Mrs. V’s right of self-determination [35, 36].

The arguments in favour of the hospital are as follows. First, although Mrs. V’s refusal to undergo blood transfusions was clearly in keeping with the legal right of bodily self-determination, Mrs. V’s attending and other hospital physicians believed that they had exhausted all non-transfusion alternatives. Based on reasonable medical certainty, it was essential that she receive blood transfusions to survive. Second, Mrs. V was young, previously healthy, and had a favourable chance of recovery from the complication; therefore, the physicians’ ethical code did not permit them to stand by idly and allow her to die. The hospital had an interest in ensuring that the ethical integrity of the medical profession remained intact. Third, the trial court, relying on the state’s interests in preserving life and protecting innocent third parties such as the baby, granted the injunction.

Arguments presented against the hospital are as follows. First, physicians should respect the decisions made by informed and competent patients. The hospital’s repudiation of the releases signed by Mrs. V constituted a breach of the hospital’s contractual duty to her. Second, practitioners of Jehovah’s Witnesses believe that although a blood transfusion might save their corporeal life, it will deprive them of eternal salvation. Therefore, a non-consensual blood transfusion is a gross physical violation as well as a violation of the individual’s values. Third, when the court request was filed by Dr. C, Mrs. V was no longer pregnant. Consequently, while concern for the long-term welfare of the baby is certainly commendable, Mrs. V’s decision to refuse a blood transfusion posed no risk to the baby’s physical health.

The issue presented in this case was of public importance, as the appeal had a significant impact on more than 23,000 Jehovah’s Witnesses residing in Connecticut at that time [35]. In this case, the Connecticut Supreme Court provided further judicial support to the right to self-determination. It was the first time that the Connecticut Supreme Court held that a medical patient, whose life was in danger but who could be cured by a simple and relatively routine treatment, had the right to refuse that treatment [36].

#### China versus the United States from a cultural perspective

The ancient Chinese Empire was a society characterized by class, patriarchy, and paternalism [37]. Chinese cultural and moral traditions shaped by Confucianism are customarily described as communitarian, familial, and patriarchal, emphasizing the importance of family, community, and state, rather than the individual. Family is the core of Chinese culture. In ancient Chinese society, the family, which is bound by blood ties, participated in social and economic activities as a basic unit. In Chinese culture, the merits of group belonging include enhancing the sense of obligation, deepening feelings, and promoting harmony among family members. Group autonomy and shared decision-making can promote these merits. Chinese culture encourages people to protect patients from mental stress and to avoid negative topics, such as informing patients of potential risks prior to surgery or telling a cancer patient the truth. In addition, patients in China may prefer to rely on their family for life care and economic support. Hence, the importance of protecting patients from mental stress and fulfilling family members’ familial obligations exceeds respect for patient autonomy. The consent of a patient’s family substitutes the principle of informed consent and autonomy of patients in Chinese medical practice.

In striking contrast to China, the concept of autonomy is a manifestation of Western culture, which emphasizes individualism. Self-determination is paramount in the liberal Western tradition that advocates for the importance of individual freedom and choice [38]. Moreover, unlike most of the population in China, which holds no religious belief, such beliefs are deeply respected in the US and play an important role in shaping a person’s value system and provide meaning in their life [39]. Consequently, the United States applies a patient-centred approach, wherein physicians are required to discuss information relevant to patients’ decisions to facilitate their autonomy [40]. Advance directives or appropriate surrogates are consulted to make decisions only when patients lack the capacity to make informed decisions.

#### China versus the United States from a legislative perspective

Schloendorff v. Society of New York Hospitals (1914) is widely regarded as a landmark in the history of informed consent and sets a precedent for bioethical autonomy [41]. The decades since the 1950s have seen an increased emphasis on the patient’s right to accept or decline recommended treatments [40]. The American Hospital Association published the Patient Bill of Rights in 1972, which focused on improving standards for respecting patients admitted to hospitals. In 1990, the US Congress passed the Patient Self-Determination Act to inform patients of their rights to decide their own medical care and to ensure that these rights are communicated to them by the health care provider.

In 2016, Taiwan passed the Patient Autonomy Act, the first law in Asia that focused on the patients’ right of autonomy. Mainland China does not have a law protecting patient autonomy; instead, it regulates informed consent and autonomy issues through specific laws, such as the Tort Liability Law, Law of Medical Practitioners, and the Administrative Regulations on Medical Institutions. These laws and regulations stipulate that informed consent forms required for medical procedures and treatments (such as surgeries) can be signed either by patients or by their relatives. However, many problems exist in the current legal system, such as the dispersion and generalization of laws, and the unification of different subjects. The Civil Code, which took effect on January 1, 2021, will provide a legislative reference for patients’ informed consent and autonomy mentioned in the clauses of tort liabilities [33].

## Conclusions

Ethical dilemmas in China have often occurred due to the late development of bioethics in the country. However, the presence of earlier bioethics in the US has not freed the country of ethical dilemmas. Culture and religion mould people’s beliefs, values, expectations about health, the physician–patient relationship, the style of decision-making and the country’s legislation. Regarding privacy, in Case 1, private body parts were infringed upon and the compensation was minimal; in Case 2, digitally altered images and sound were made public and the fine was immense. Regarding autonomy, in Case 3, the subject of informed consent was the patient’s family. The hospital did not perform the surgery due to her family’s refusal, and the patient died, however, it was ruled that the hospital had not caused any infringement. In Case 4, the subject of informed consent was the patient. The hospital prescribed a blood transfusion which the patient rejected; the patient survived, but it was ruled that the hospital violated the patient’s right of self-determination. The extent to which privacy and autonomy were valued and enforced by the courts differed in China and the United States. A comparative analysis of the cases referenced provides insight to understanding the practice of bioethics from both an intercultural and a legislative perspective.

Because China has a profound cultural tradition, the tension between international ethical norms and local culture must be handled with care. On the one hand, for autonomy, the core and essence of ethical standards of informed consent must be adhered to, while the relevant methods and processes can be altered according to cultural backgrounds and/or specific situations. On the other hand, for privacy, positive values and beliefs must be respected and sustained, while obsolete concepts that have exerted negative influences must be discarded. In terms of protecting patients’ rights and handling ethical problems, Western countries have a more developed system than China; therefore, owing to their practical experience, they can serve as a legislative reference for China to develop a more advanced concept of bioethics that incorporates modern features to meet future requirements.

## Data Availability

Not applicable.

## Abbreviations

NYP: NewYork-Presbyterian
ABC: The American Broadcasting Company
OCR: Office for Civil Rights
HIPAA: Health Insurance Portability and Accountability Act

## References

[1] Whitehouse PJ (2003). The rebirth of bioethics: extending the original formulations of Van Rensselaer Potter. Am J Bioeth.

[2] Chadwick R, Wilson D (2018). The emergence and development of bioethics in the UK. Med Law Rev.

[3] Li EC (2008). Bioethics in China. Bioethics.

[4] Hain R, Saad T (2016). Foundations of practical ethics. Medicine.

[5] Piccoli GB, Sofronie AC, Coindre JP (2017). The strange case of Mr. H. Starting dialysis at 90 years of age: clinical choices impact on ethical decisions. BMC Med Ethics.

[6] Lawrence DJ (2007). The four principles of biomedical ethics: a foundation for current bioethical debate. J Chiropr Human.

[7] Beauchamp TL, Childress JF (2013). Principles of biomedical ethics.

[8] Gillon R (1994). Medical ethics: four principles plus attention to scope. BMJ.

[9] Scott PA, Välimäki M, Leino-Kilpi H, Dassen T, Gasull M, Lemonidou C (2003). Autonomy, privacy and informed consent 1: concepts and definitions. Br J Nurs.

[10] Mohammadi M, Larijani B, Emami Razavi SH, Fotouhi A, Ghaderi A, Madani SJ (2018). Do patients know that physicians should be confidential? Study on patients' awareness of privacy and confidentiality. J Med Ethics Hist Med.

[11] Dickenson D, Huxtable R, Parker M (2010). The Cambridge medical ethics workbook.

[12] Faria MA (2015). Religious morality (and secular humanism) in Western civilization as precursors to medical ethics: a historic perspective. Surg Neurol Int.

[13] Berman HJ (1974). The interaction of law and religion.

[14] Wang R, Henderson GE (2008). Medical research ethics in China. Lancet.

[15] Bowman KW, Hui EC (2000). Bioethics for clinicians: 20. Chin Bioeth CMAJ.

[16] DuVal G, Clarridge B, Gensler G, Danis M (2004). A national survey of U.S. internists' experiences with ethical dilemmas and ethics consultation. J Gen Intern Med..

[17] AbuAbah F, Alwan A, Al-Jahdali Y, Al Shaikh A, Alharbi A, Al-Jahdali H (2019). Common medical ethical issues faced by healthcare professionals in KSA. J Taibah Univ Med Sci.

[18] Tripathi R, Ezaldein HH, Rajkumar K, Bordeaux JS, Scott JF (2019). Characteristics of state and federal malpractice litigation of medical liability claims for keratinocyte carcinoma, 1968 to 2018. JAMA Dermatol.

[19] Annas GJ (1984). The case of Baby Jane Doe: child abuse or unlawful Federal intervention?. Am J Public Health.

[20] Fine RL (2005). From Quinlan to Schiavo: medical, ethical, and legal issues in severe brain injury. Proc (Bayl Univ Med Cent).

[21] Maclean A (2009). Autonomy, informed consent and medical law: a relational challenge.

[22] Zhao YR, Liu C. Hospitals' teaching task and the protection of patients' privacy rights. Med Philos. 2007;28(4):29–30+40 (**in Chinese**).

[23] Ahmed Mel-B K (2002). What is happening to bedside clinical teaching?. Med Educ..

[24] Wolfberg AJ (2007). The patient as ally–learning the pelvic examination. N Engl J Med.

[25] Rakatansky H (2016). Filming in the ED: a cautionary tale NY hospital settles $2.2M claim over patient-privacy violations suit. R I Med J (2013).

[26] Blain B. Judge scrubs suit accusing ABC of privacy violations after network aired footage of dying man on 'NY Med' reality series. New York Daily News; 2016. https://www.nydailynews.com/new-york/judge-scrubs-suit-abc-ny-med-privacy-violations-article-1.2584257. Accessed 3 Jan 2020.

[27] Ornstein C: When a patient's death is broadcast without permission. ProPublica; 2015. https://www.propublica.org/article/when-a-patients-death-is-broadcast-without-permission. Accessed 3 Jan 2020.

[28] Chanko P. Loved ones dying on TV, without consent: New York needs laws that prevent filming in hospitals without patients' permission. New York Daily News; 2016. https://www.nydailynews.com/opinion/pamela-chanko-loved-dying-tv-consent-article-1.2578097. Accessed 3 Jan 2020.

[29] Nie JB (2011). Medical ethics in China: a transcultural interpretation.

[30] Warren SD, Brandeis L (1890). The right to privacy. Harvard Law Rev.

[31] Head T. Where did the right to privacy come from? ThoughtCo; 2019. https://www.thoughtco.com/right-to-privacy-history-721174. Accessed 23 Mar 2020.

[32] Cohen IG, Mello MM (2018). HIPAA and protecting health information in the 21st century. JAMA.

[33] China Today. China's Civil Code a landmark law to protect people's rights. China Today; 2020. http://www.chinatoday.com.cn/ctenglish/2018/hotspots/2020lianghui/com/202005/t20200529_800207991.html. Accessed 30 May 2020.

[34] Li EC, Wen CF (2010). Should the Confucian family-determination model be rejected? A case study. J Med Philos.

[35] Connecticut. Supreme Court. Stamford Hospital v. Vega. Atl Report. 1996;674:821–34. 11648307

[36] Hankins JM (1996). The common law right of bodily self-determination in Connecticut: life and death after Stamford Hospital v. Vega. Conn Law Rev.

[37] Tsai DF (1999). Ancient Chinese medical ethics and the four principles of biomedical ethics. J Med Ethics.

[38] Will JF (2011). A brief historical and theoretical perspective on patient autonomy and medical decision making: Part II: the autonomy model. Chest.

[39] Martin AM (2007). Tales publicly allowed: competence, capacity, and religious belief. Hastings Cent Rep.

[40] Krumholz HM (2010). Informed consent to promote patient-centered care. JAMA.

[41] Chervenak J, McCullough LB, Chervenak FA (2015). Surgery without consent or miscommunication? A new look at a landmark legal case. Am J Obstet Gynecol.

